# Utilizing digital technologies to promote well-being in university students: the ‘DigiWell’ research protocol

**DOI:** 10.3389/fpsyg.2024.1397870

**Published:** 2024-09-05

**Authors:** Chiara Ruini, Francesca Vescovelli, Valentina Paola Cesarano, Grazia De Angelis, Erika De Marco, Elisabetta Lucia De Marco, Gian Maria Galeazzi, Giorgio Li Pira, Luca Pingani, Pierpaolo Limone

**Affiliations:** ^1^Department of Life Quality Studies, University of Bologna, Rimini, Italy; ^2^Department of Psychology and Education, Pegaso University, Naples, Italy; ^3^Department of Biomedical, Metabolic and Neural Sciences, University of Modena and Reggio Emilia, Reggio Emilia, Italy; ^4^Department of Integrated Activities in Mental Health - Pathological Addictions, Azienda USL – IRCCS di Reggio Emilia, Reggio Emilia, Italy; ^5^Department of Psychology, University of Bologna, Bologna, Italy

**Keywords:** university students, mental health, well-being, positive psychology, virtual reality, positive interventions

## Abstract

The COVID-19 pandemic has significantly impacted the well-being of university students, particularly those in healthcare and medical programs. Psychological interventions rooted in positive psychology have proven effective in enhancing mental health, with online and digital delivery methods proving to be equally viable. This study aims to enhance mental health among Italian university students through digital interventions, including virtual reality, utilizing a stepped care approach. Specific objectives include implementing online positive interventions for students experiencing mild distress (DigiWell_Step 1), evaluating a Virtual-Reality intervention for moderate distress (DigiWell_Step 2), and identifying students experiencing high distress or optimal well-being. Cluster analyses and linear models will assess intervention outcomes. It is anticipated that students undergoing these steps will experience significant improvements in well-being and distress reduction, with sustained benefits at a three-month follow-up. This research contributes to understanding the efficacy of mental health interventions for university students, leveraging digital technologies to enhance accessibility and user engagement. The integration of digital technologies enhances the cost-effectiveness and engagement of interventions delivered through a stepped care approach tailored to the targeted population.

## 1 Introduction

The Covid 19 pandemic, spanning over two academic years, has had a detrimental impact on the quality of life and well-being of university students (Li et al., 2021; Zarowski et al., 2024). Globally, numerous campuses closed their doors, transitioning courses to online platforms (Toto and Limone, 2021a). Mental health issues among students have been attributed to concern about contracting the virus and diminishing social support. Cao et al. (2020) found that 24.9% of college students experienced elevated levels of anxiety due to the impact of COVID-19 on their academic activities, daily lives (such as social distancing), and economic prospects. Students enrolled in healthcare or helping profession courses appear to be particularly vulnerable to anxiety and depression. Recently, Nayak et al. (2021) found that the estimated rates of depression and anxiety were highest among veterinary students, followed by dental, medical and pharmacy students. The nursing student population, which represents the largest cohort within the health professions, reported high rates of depression (30%), anxiety (38%), and traumatic stress (30%) (Murat et al., 2021; Jardon and Choi, 2022). A recent study suggested that college students’ levels of anxiety, depression, and stress might be associated with lower levels of empathy and difficulties in emotion regulation (MacDonald and Price, 2019). Specifically, individuals with anxiety and/or depressive disorders exhibited poor understanding of emotions and their components, a high tendency to react negatively to emotional experiences, and difficulties in recovering from negative emotions (Mennin et al., 2009). Moreover, college students and young adults were found to be reluctant to seek psychological help due to general misinformation about mental health and the fear of being stigmatized (Eisenberg et al., 2007; Eisenberg et al., 2009a).

For these reasons, MacDonald and Price (2019) suggested that the development and practice of emotion regulation skills in college students may represent a preventive intervention for enhancing students’ mental health. Furthermore, better education on mental health in younger generations can significantly reduce the risk of perceived stigma (Pingani et al., 2021) and foster a better understanding of the components and characteristics of mental health *vs* mental illness.

The current definition of mental health, in fact, posits that it is not merely the absence of psychopathology; rather, it involves the presence of positive characteristics such as well-being, positive relationships and social contribution (Keyes, 2002; Ruini, 2017). According to Keyes’ conceptualization, well-being is an essential contributor to individuals’ mental health. He postulated the *Mental Health Continuum* (MHC), where high levels of well-being correspond with complete mental health (also known as the state of *flourishing*), characterized by positive feelings and good social and psychological functioning. Individuals on the “incomplete” side of the spectrum are categorized as *languishing* (i.e., more vulnerable to mental illnesses), whereas those whose mental health tends to fall in between are referred to as having *moderate mental health*, thus presenting impairments in some dimensions of well-being (Keyes, 2002). Scientific evidence has suggested that high levels of flourishing may protect against mental illness, whereas languishing may represent a risk factor for mood and anxiety disorders (Iasiello et al., 2019; Keyes et al., 2010).

Various investigations have explored flourishing, languishing and moderate mental health among university students. Keyes et al. (2012) found that the proportion of students classified as flourishing, moderate, and languishing mental health was 51.8, 44.6, and 3.6%, respectively. Similarly, Fink (2014) found that 59.2% of students were flourishing, 39.5% were moderately mentally healthy, and 1.3% of students were languishing. In all these investigations, only the condition of flourishing was associated with better academic performance, higher levels of school engagement, and lower rates of mental illnesses.

Recent studies have documented that flourishing mental health in college students was negatively associated with fear of Covid-19 contagion, financial insecurity due to the pandemic, social distancing and online participation in teaching activities (Elemo et al., 2021). In Italy, Capone et al. (2020) documented that during the pandemic, 17.5% of the students were languishing, 52.9% were moderately mentally healthy, and 22.3% were flourishing. Those who were flourishing reported using social media and other digital technologies to stay informed during the lockdown and perceived a lower risk to their health.

Indeed, research has shown that digital media may enhance personal well-being when they support basic processes such as active participation, connection to the real world, facilitation of teamwork, and the increase of intrinsic motivation. Digital media, therefore, can improve academic success and student’s well-being if properly used (Toto and Limone, 2021b).

During the past 2 years, digital technologies have become essential tools also for delivering psychosocial interventions to reduce the psychological burden of the pandemic and to help individuals flourish even during difficult and complex times (Riva et al., 2020; Yotsidi et al., 2023; Abulfaraj et al., 2024). As a response to the psychological distress caused by the pandemic, university worldwide have increased the delivery of psychological counseling to support their students (Romeo et al., 2021). Moreover, many universities (including Italian ones) have implemented additional digital tools to sustain students’ wellbeing, foster their resilience, and promote better emotion regulation strategies (Di Consiglio et al., 2021b; Di Consiglio et al., 2021a).

A recent review (Li Pira et al., 2023) confirmed the beneficial effect of using virtual reality when providing psychological treatment for various mental health conditions (i.e., anxious and mood disorders; eating disorders, psychosis, obsessive compulsive disorders etc.) Indeed, some transdiagnostic VR software protocols have been found to ameliorate emotion regulation (ER) strategies in the general population (Gardini et al., 2023) and in young adults (Pancini et al., 2022). However, according to this recent review article (Li Pira et al., 2023) no VR software or intervention has been specifically designed to promote the use of psychological resources and to address the complete spectrum of mental health (Mennin et al., 2009). Moreover, the beneficial effects of VR-based interventions, including the improvement in the perception of stigma and the promotion of psychological well-being, have not yet been proven.

Building on this initial state of the art, the present research aims to develop an intervention protocol for university students experiencing psychological distress using a VR software and other digital technologies. The study aims to improve students’ emotion regulation strategies, their perception of mental health stigma, and their levels of psychological well-being. The description of this new protocol will be provided below.

The study will be longitudinal and will consist of different phases: (1) the screening phase, during which participants will be enrolled and selected based on their profile of psychological well-being and distress; (2) the intervention phase, during which students will be allocated to receive either a group intervention delivered online via the ZOOM platform (DigiWell_step 1) or an individual psychological intervention integrated with the use of Virtual Reality software (DigiWell_step 2); (3) the follow-up phase, where assessment will be repeated 3 months after the conclusion of the interventions.

The hypotheses are that the DigiWell interventions will prove to be effective in reducing distress as well as improving psychological well-being, from pre- to post-intervention and that these results will be maintained at the three-month follow-up.

## 2 Method

### 2.1 Recruitment of participants and study design

Three Italian Universities developed the current research project and received a national grant for its implementation. Two universities are public (one large and the second medium-sized), while the third is private, with various faculties across the country. All universities are located in urban area. The research project will be promoted through local seminars and on the webpages and social media of the three institutions. Recruitment of students will focus on those enrolled in university courses related to health and helping professions. A minimum of 300 university students from the three universities (100 students per institution) will be recruited to participate on a voluntarily basis. Students will be contacted via email and receive a digital link to an online platform, which will also be shared on the main social media channels and social networks of the universities.

**Figure 1:**
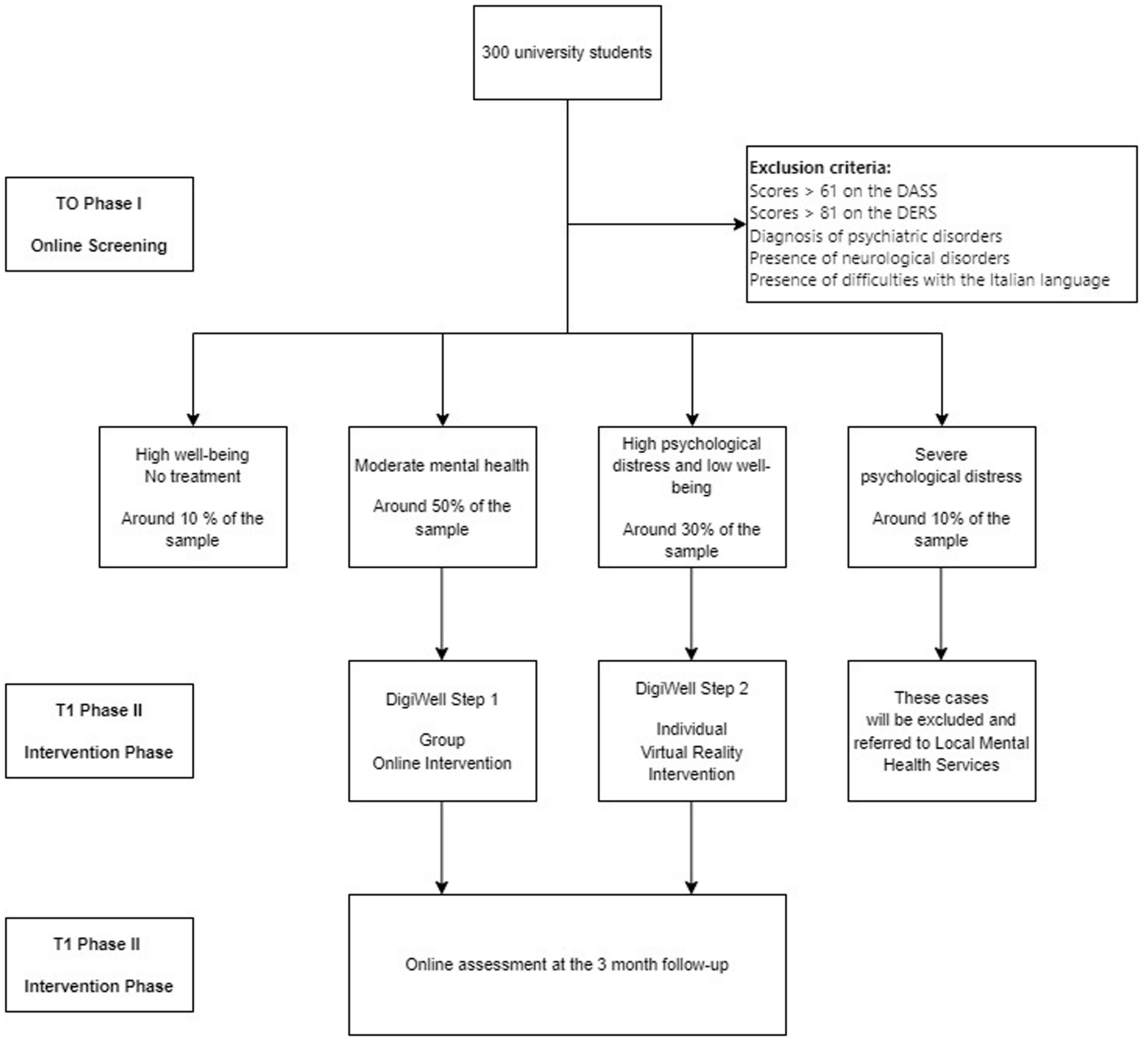
Research protocol flowchart.

Within this link, participants will read a description of the project consisting of three phases: screening, intervention and follow-up. Only those who will accept to participate in the project and will sign the written informed consent online will be included in the sample. They will then gain access to the screening phase (see section below and Figure 1 flowchart).

The screening will be conducted via an online platform (Qualtrics), where specific questions on sociodemographic factors (age, gender, income, marital status, levels of education, university course attendance) and clinical characteristics (presence of psychiatric disorders, neurological disorders, or other medical conditions that might hinder the use of digital technologies and virtual reality devices) will be presented. Additionally, students will be asked to complete specific psychometric questionnaires: the Depression Anxiety Stress Scale (DASS-21) (Bottesi et al., 2015), the Difficulties in Emotion Regulation Scale (DERS) (Sighinolfi et al., 2010), and the Mental Health Continuum (MHC) (Petrillo et al., 2014) for assessing well-being and mental health (see the next paragraph for description of the questionnaires).

Based on students’ scores on these questionnaires, they will be assigned to either the group intervention DigiWell_Step 1 or the individual VR intervention DigiWell_Step 2.

*The inclusion criteria for DigiWell_Step 1 are as follows*: (1) presence of mild levels of psychological distress, as assessed through DASS (total score ranging from 10 to 30); (2) presence of mild impairments in well-being, as assessed through the MHC (students categorized in the Moderate Mental Health group); (3) presence of mild emotional dysregulation, as assessed through the DERS (total score ranging from 36 to 60). At least one of those criteria must be met. Participants meeting the inclusion criteria will be contacted via phone or email and invited to participate in the intervention phase of the project, where they will receive the online group intervention aimed at promoting well-being and minimizing mental health stigma.

*The inclusion criteria for DigiWell_Step 2 are as follows*: (1) presence of moderate to severe psychological distress, as assessed through DASS (total score ranging from 31 to 60); (2) presence of severe impairments in well-being, as assessed through MHC (students categorized in the Languishing group); (3) presence of clinically significant emotional dysregulation, as assessed through DERS (total score ranging from 61 to 80); (4) absence of clinical conditions that would hinder the use of virtual reality devices. At least one of those criteria must be met. Participants meeting the inclusion criteria will be contacted via phone or email and invited to participate in the intervention phase of the project, where they will receive the individual psychological intervention integrated with the use of a Virtual Reality software (DigiWell_ step 2).

The exclusion criteria from the intervention phase of the project will be as follows: (1) presence of clinically severe levels of psychological distress (DASS total score > 61); (2) presence of severe emotional dysregulation (DERS total score > 81); (3) presence of a psychiatric diagnosis of bipolar disorder, schizophrenia, psychotic disorder, or personality disorder (as indicated in clinical data from the online screening); (4) presence of neurological disorders; (5) refusal to sign the informed consent; or (6) difficulties in understanding and producing the Italian language.

Participants who will exhibit high levels of psychological distress (DASS score > 60), and/or report the presence of a psychiatric condition, and/or demonstrate severe emotional dysregulation (DERS scoring >81) will be contacted and encouraged to seek assistance from local mental health centers or to the university counseling service to receive tailored psychotherapeutic or pharmacological treatments.

Furthermore, participants who will not exhibit psychological distress during the initial screening (DASS scoring <10), and/or will not report impairments in wellbeing (those categorized in the flourishing group of the MHC questionnaire), and/or demonstrate effective emotional regulation strategies will be excluded from the intervention phase of the project. However, their socio-demographic data and clinical features of their flourishing will be analyzed.

This meticulous screening procedure will enable an initial assessment of the mental health status of university students, distinguishing resilient individuals with good mental health, (thus not requiring psychological intervention), from those displaying specific vulnerabilities. These vulnerable students will be assigned to either DigiWell_Step 1 or Step 2 interventions aimed at restoring their levels of well-being.

### 2.2 Procedure

Participants meeting the criteria for DigiWell Step 1 or DigiWell Step 2 will be subsequently contacted via e-mail or smartphone.

All interventions will be conducted via telemedicine/telepsychology, utilizing digital technologies such as videoconference platforms and, for DigiWell_Step 2 intervention only, virtual reality headset.

### 2.3 Interventions

In line with previous meta-analyses (Van Agteren et al., 2021; Bolier et al., 2013) that have shown more beneficial effects on well-being for interventions of high intensity (i.e., longer that 1 month) and including multiple components of well-being, our interventions will consist of 6 sessions covering different positive dimensions. Both interventions will be delivered according to specific written manuals detailing various activities and topics to be covered by therapists (psychologists, psychotherapists and psychiatric rehabilitation technicians) or group leaders (Pingani et al., 2013). Treatment fidelity will be ensured through supervision to maintain protocol adherence, and sessions will be recorded randomly.

#### 2.3.1 DigiWell_Step 1

Group intervention for wellbeing promotion, previously validated on students and young individuals (Tomba et al., 2010; Ruini et al., 2006; Ruini et al., 2009). This intervention will comprise six 2-h group sessions conducted weekly via the Zoom or TEAMS digital platform. Each group, consisting of 15–20 students recruited from participating universities, will be led by a psychologist trained in well-being promoting strategies. Sessions will cover topics related to positive emotions and dimensions of wellbeing, with participants engaging in discussion, role-playing, and sharing happiness and well-being activities. Each session will address different dimensions of well-being. The first session will focus on positive affectivity, involving activities such as recalling and sharing situations that evoke positive emotions and short exercises of savoring meditation. Another session will explore purpose and meaning in life, including discussions on hope and goal pursuits, where participants will share personal goals for the future and identify pathways to achieve them. Two sessions will concentrate on identifying participants’ personal resources and character strengths, emphasizing self-acceptance. One session will cover positive interpersonal relationships and social contribution. Participants will identify significant people in their lives (partners, family members, and friends) and share positive emotions and events with them. This session will also include reflections on interpersonal forgiveness and gratitude dimensions. Additionally, a session dedicated to mental health discrimination will be included. It will focus on the concept of stigma, providing a definition (Goffman, 1961), and will address different types of stigma in mental and general health (Pingani et al., 2021; Evans-Lacko et al., 2016), as well as its working and social consequences (Evans-Lacko et al., 2016; Pingani et al., 2016; Boudewyns et al., 2015).

#### 2.3.2 DigiWell_Step 2

Psychological intervention involving six individual sessions integrated with a virtual reality software. This software will simulate environments where students typically spend their time, such as a studio, house, bathroom, and bedroom. Participants will interact with various objects and stimuli within each environment (food, drinks, a pack of cigarettes, a videogame console, and a smartphone), identifying stressful/unpleasant situations and practicing emotion regulation strategies. The virtual house will contain a “resources box” with psychological resources for well-being (interpersonal relationships, self-acceptance and self-esteem, but also sports, leisure and artistic activities, life goals and meaning). Participants will be asked to explore and select resources according to their needs and preferences. The aim will be to assist participants in reflecting and exploring the available resources for personal well-being within the virtual resources box. The clinician will aim to utilize the DigiWell_step 2 software to encourage patients to adopt new coping strategies for their difficulties, subsequently applying these newfound skills in real-world situations. Similarly, therapist working with participants will undergo through training and supervision in software usage, following a detailed manual outlining session description and targeted activities for implementation with the virtual reality software. Therapists will facilitate discussion on the virtual experience and devise strategy for implementing changes in participants’ real-life contexts. Additionally, DigiWell_step 2 intervention will incorporate a specific form within each session to provide participants with information and knowledge about common mental health problems, thereby enhancing their subjective perception of stigma.

### 2.4 Measures

#### 2.4.1 T0 (initial screening)

Participants providing written informed consent and meeting the study criteria will be asked to complete the following questionnaires online via the Qualtrics platform at multiple time points:

- A non-psychometric self-report survey to collect socio-demographic data (age, marital status, educational level, occupational status) and clinical information (medical chronic conditions, former DSM-5-TR diagnosis, clinical conditions, and/or use of medical devices interfering with VR), as well as to assess participants’ familiarity with the use of computer or technological devices (e.g., computers, video games) in their daily lives.
- Depression Anxiety Stress Scales (DASS-21) (Bottesi et al., 2015; Lovibond and Lovibond, 1995): a self-report questionnaire comprising 21 items measuring depression (7 items), anxiety (7 items), and stress (7 items). The scores on the 3 subscales will be summed to provide an overall assessment of individual psychological distress. Participants will rate each item on a scale from 0 (did not apply to me at all) to 3 (applied to me very much). Total scores range from 0 to 120, with subscales scores ranging from 0 to 42. The Italian validation of the questionnaire demonstrated good psychometric properties. Clinical cut-off scores have been established: scores ≥60 and ≥ 30 for the DASS-total are labeled as “high” or “severe” and as “mild” or “moderate,” respectively (Lovibond and Lovibond, 1995; Beaufort et al., 2017);
- Difficulties in Emotion Regulation Scale (DERS-36) (Sighinolfi et al., 2010): a 36-item scale designed to assess various aspects of emotion regulation, including the ability to identify, differentiate, and accept emotional experiences, engage in goal-directed behavior, inhibit impulsive behavior in negative emotional contexts, and use effective emotion modulation strategies. Respondents will rate each item on a 5-point Likert scale ranging from 1 (not at all) to 5 (completely), with total scores ranging from 36 to 180. Higher scores indicate greater emotional dysregulation. Prior research has suggested that a score of 62 was capable of predicting generalized anxiety disorders in a sample of community dwellers, while the clinical range for the DERS total score typically falls between averages of approximately 80 to 127 (Mennin et al., 2009; Staples and Mohlman, 2012). The DERS has demonstrated good psychometric properties, including in its Italian validation (Sighinolfi et al., 2010);
- Mental Health Continuum Short Form (MHC-SF) (Petrillo et al., 2014; Keyes, 2002): a 14 items scale assessing emotional well-being (items 1–3), social well-being (items 4–8), and psychological well-being (items 9–14). Participants indicate how frequently they experienced each symptom of mental well-being over the past month, ranging from 0 (none of the time) to 5 (all of the time). Scores are totaled to provide a quantitative indicator of mental health, with a range of 0–70. Additionally, Keyes’ categorical diagnosis can be used to cluster participants according to their levels of well-being. The diagnosis of flourishing can be made if a participant rates at least one of the three emotional well-being items “every day” or “almost every day,” and at least six of the 11 psychological and social well-being items as “every day” or “almost every day.” The diagnosis of languishing can be made if participant rated at least one of the three emotional well-being items as “never” or “once,” and at least six of the 11 psychological and social well-being items as “never” or “once.” Participants who neither qualify as flourishing nor languishing are categorized as moderately mentally health. The instruments have demonstrated good psychometric properties in several international validation studies, including those conducted in Italy (Petrillo et al., 2014; Keyes, 2002).

#### 2.4.2 T1 (pre-intervention assessment)

Before being allocated either to Digiwell_Step 1 or Digiwell_Step 2 according to the screening procedures, all participants enrolled in the study should complete these additional questionnaires:

- Psychological Well-Being Scales (PWB) (Ryff, 1989; Ruini et al., 2003) is a 42 item self-rating inventory that covers the six areas of psychological well-being: autonomy (7 items), environmental mastery (7 items), personal growth (7 items), positive relations with others (7 items), purpose in life (7 items), and self-acceptance (7 items). Participants respond on a 6-point format ranging from “strongly disagree” to “strongly agree.” Responses to negatively scored items will be reversed in the final scoring on the assessed dimension. A total PWB score can be calculated by adding together the scores of the six dimensions, with a range of 42–252. The Italian version of PWB scales has satisfactory test–retest reliability (ranging between 0.81 and 0.88 in a six-week interval) and is inversely related to measures of psychological distress (Brandel et al., 2017).
- Positive and Negative Affect Schedule (PANAS) (Watson et al., 1988; Terraciano et al., 2003) is the most frequently used instrument to assess positive affect (10 items) and negative affect (10 items). The Positive Affect scale reflects the level of pleasant engagement, the extent to which a person feels enthusiastic, excited, active, and determined. The Negative Affect scale reflects a general dimension of unpleasant engagement and subjective distress that subsumes a broad range of aversive affects including fear, nervousness, guilt, and shame. Participants indicate the extent they have felt the 20 emotions over the past week, using a Likert scale from 1 (not at all) to 5 (extremely), with a scale score ranging from 10 to 50. The PANAS scales have shown excellent psychometric properties (reliability, convergent and divergent validity) and have been translated into several languages, including Italian (Terraciano et al., 2003).

Stigma will be assessed by:

- The Italian version of the Mental Health Knowledge Schedule (MAKS-I) (Pingani et al., 2019), a self-administered questionnaire composed of 12 items scored on a Likert scale (from 1: “Strongly Disagree” to 5: “Strongly Agree”). “Do not know” is coded as neutral (value of 3) according to the scoring guidelines. The MAKS-I questionnaire is categorized into two parts. The first six statements can be summed into a total score representing stigma-related mental health knowledge (the higher the score, the greater the knowledge of mental illness). Items from 7 to 12 assess recognition and familiarity with six different conditions.
- The Italian version of the Attribution Questionnaire 26 (AQ-27-I) (Pingani et al., 2012), a self-administered questionnaire. Respondents will be asked to rate their level of agreement with 27 statements about “Harry,” a 30-year-old single man with schizophrenia, on a Likert scale from 1 (not at all) to 9 (very much). The AQ-27-I includes nine subscales, each assessing a typical stereotype about people with mental illness: responsibility, pity, anger, dangerousness, fear, help, coercion, segregation, and avoidance. Higher scores indicate greater stigmatization toward “Harry” (in the Italian version, the subscales for help and avoidance are reverse scored).

#### 2.4.3 T2 and T3 (post-intervention and follow-up)

At the end of the interventions (T2) and after 3 months (T3) participants will be assessed with the full battery of questionnaires, including those administered in the screening phase (T0).

### 2.5 Statistical analysis

A convenience sample of 300 university students will be used for the statistical analyses. For the screening phase, the sociodemographic, clinical, and anamnestic data will be analyzed through descriptive statistics (frequency, percentile, mean scores, cutoff scores, etc.).

Using K-mean cluster analysis, different subgroup of students will be identified based on the categories of mental health resulting from the standardized score of DASS, DERS, and MHC. Four group will be calculated: (1) Moderate well-being (presence of mild levels of psychological distress, mild impairments in well-being, and mild emotional dysregulation); (2) Languishing well-being (presence of moderate levels of psychological distress, moderate to high emotional dysregulation, and significant impairments of well-being); (3) Distress group (presence of clinically significant levels of psychological distress, severe emotional dysregulation, and absence of well-being: these students will be considered as presenting clinically impaired mental health); (4) Flourishing well-being (diagnosis of flourishing at MHC, low levels of psychological distress, and absence emotional dysregulation). These students will be considered as presenting optimal mental health.

Descriptive statistics for the clusters will be performed, and Chi-square and General Linear Model will be used to compare the clusters of participants.

In the intervention phase, pre-to-post changes in DASS, MHC, and DERS mean scores will be considered as primary outcomes for both interventions, while pre-to post changes in PANAS, PWB (well-being), and in MAKS-I and AQ (stigma) will be considered as secondary outcomes.

To control between-group variance due to group belonging (Digiwell_step 1 and Digiwell_step 2), variance component analyses and intraclass correlation values (ICC) will be calculated for secondary outcome measures (total scores on PWB; PANAS, MAKS-I). Additionally, baseline differences among the groups will be calculated using MANOVA, with group assignment as fixed factor and secondary outcome measures as dependent variables.

In the follow-up phase, the specific effects of the stepped care approach (DigiWell_Step1 and Digiwell_Step2) will be analyzed with two separated repeated-measure multivariate analyses, with time (3 levels pre-post-follow up) as between-subject factor and using contrast analysis between baseline score — post-intervention and follow-up mean scores. In both interventions, primary outcomes (dependent variables) will be DASS, DERS, and MHC total scores. Secondary outcomes will be PWB, PANAS, and STIGMA total scores, and they will be used as dependent variables in the multivariate models.

The partial eta-squared, as a measure of effect size, will be also calculated considering a value of 0.1 as a large effect, a value of 0.04 as a medium effect, and a value of 0.01 as a small effect (Huberty, 2002). The significance level was set at *p* < 0.05. Data analyses will be performed using SPSS Statistic 28.

### 2.6 Ethical statement

This study has been approved by the Ethics Committee of the University of Bologna (Protocol No. 0388152, dated 28/12/2023) and has also been ratified by the Ethics Committee of the University of Modena and Reggio Emilia (UNIMORE) (Protocol No. 24/02/2024). Anonymity of participants from T1 to T2 and T3 will be safeguarded through strict data handling protocols, including anonymization of collected data and secure storage measures.

## 3 Expected results

It is expected that students treated with DigiWell_Step 1 e Step 2 will significantly improve their level of wellbeing (MHC as primary outcome, PWB and PANAS as secondary outcome) and at the same time, they will decrease their psychological distress measured by DASS (primary outcome) and PANAS (negative emotions as secondary outcome) at post-intervention. Furthermore, it is expected that participants will improve their strategies of emotional regulation (decrease of DERS scores) and their perception of stigma toward mental health (MAKS-I, AQ-27). Moreover, it is expected that the improvements at post-intervention will be maintained also after the three-month follow-up.

## 4 Conclusion

The university context is considered as an ideal setting to reach a large proportion of the young adult population. The project will allow categorizing the sample of the student population recruited from various Italian universities according to their levels of mental health, providing a complete continuum ranging from flourishing students to languishing students to those with severe psychological distress (Capone et al., 2020).

Through an accurate screening procedure, the project will enable the collecting of an initial picture concerning the mental health of the student population and will identify the most resilient individuals who have maintained a good mental health profile (and therefore do not need psychological intervention) from those who present specific vulnerabilities in terms of presence of psychological distress, emotional dysregulation, and impairments in their levels of psychological well-being. Based on their mental health profiles, students will be allocated to specific psychological interventions, following a stepped care approach. These interventions will aim to restore well-being, potentially preventing the onset of future psychological disorders (Petrillo et al., 2014; Bower and Gilbody, 2005).

The stepped care approach could represent an innovation in the panorama of psychological interventions in university settings, aiming to implement early, low-intensity interventions with targeted strategies, and then to intensify the clinical services provided according to the users’ specific needs (Bower and Gilbody, 2005; Cornish et al., 2017). Promoting psychological well-being could represent a protective factor for mental health (Petrillo et al., 2014; Bower and Gilbody, 2005). Impairments in mental health among university students have been associated with poor academic performance and a higher risk for dropping out before graduation (Eisenberg et al., 2009b; Lipson and Eisenberg, 2018). Thus, DigiWell project could also improve academic performance and prevent the risk of early academic leaving among university students. If the findings of the present research project confirm the beneficial effects of the digital interventions, these intervention protocols could seamlessly integrate into traditional university counseling services. These services have already embraced digital tools for delivering teaching activities and psychological interventions during the pandemic (Romeo et al., 2021; Di Consiglio et al., 2021a; Li Pira et al., 2023), emphasizing the importance of promoting students’ wellbeing as primary goal.

Moreover, the project will provide information concerning university students who have optimal levels of well-being (flourishing individuals) and do not need specific interventions to support their mental health. Understanding their socio-demographic and clinical characteristics could advance scientific knowledge on the dimensions of resilience among university students, subsequently targeting future interventions for promoting these specific dimensions.

The use of digital technologies will make the interventions provided in a stepped care approach more usable, cost-effective, and engaging for the targeted population. These psychological interventions delivered via digital technologies are expected to restore participants’ mental health. A recent review documented that VR has well-documented positive effects for treating psychological distress (Li Pira et al., 2023), but there is limited evidence on the positive effects of digital technologies in promoting wellbeing. This project aims to advance the literature by investigating whether VR and other digital tools can enhance positive emotions, psychological wellbeing, and social wellbeing in young adults. The project will allow verifying the role of digital technologies in providing well-being promoting interventions potentially significantly reducing the costs of mental health services in university settings and reaching a greater number of participants, including students who do not normally attend campuses (off-site students, working students, part-time, etc.) (Beaufort et al., 2017).

In conclusion, while this research protocol holds promise for advancing our understanding of creating a digitalized stepped care approach and identifying “best practices” for promoting the mental health of university students, it is important to acknowledge potential limitations that may arise during its implementation. Self-selection in participant recruitment could introduce bias, as individuals who choose to participate may differ systematically from those who do not, affecting the generalizability of findings. Additionally, the potentially low representativeness of the sample, consisting of students from three universities (two geographically close and one online), may limit the broader applicability of study outcomes across the entire national territory. Therefore, it is essential to apply the protocol in diverse contexts.

## Data Availability

The original contributions presented in the study are included in the article/supplementary material, further inquiries can be directed to the corresponding author.
